# Supplementary material for: Transforming medical device biofilm control with surface treatment using microfabrication techniques
Source: PLoS One. 2023 Nov 30;18(11):e0292647. doi: 10.1371/journal.pone.0292647 (PMC10688649; doi:10.1371/journal.pone.0292647)
Supplement: Attachment — Submitted filename: Response to Reviewers.docx [file pone.0292647.s001.docx]

Dear Professor Geelsu Hwang,

Thank you very much for handling the review of our manuscript titled “Revolutionizing Medical Device Infection Control with Surface Treatment Using Microfabrication Techniques” which we would like to be considered for publication in the prestigious journal, PLOS One. Please note that we took all reviewers' comments into considerations and have revised the manuscript accordingly. Below are our responses to all the issues raised by the reviewers as well as a summary of changes that have been incorporated into a revised version of our manuscript.

Reviewer #1:

Dear Authors,This is an interesting study. However, the presentation is not acceptable. Poor English and lack of scientific presentation are lowering the merits of this study. It’s not acceptable in its current form. A major revision/rewriting is required for further consideration. I have some comments below for your reference.

Simple English should be used for better understanding. Readers who are not in your field should understand your manuscripts. Please maintain consistency in your writing.

The aim and objective should be well reflected in the conclusion. Redundant words are complicating this manuscript. Please avoid the unnecessary use of adjectives. Please check your English with a native proofreader.

Response: We thank the reviewer for this feedback and for the time spent reading and evaluating our manuscript. In the revised manuscript, we simplified the language used when appropriate, reduced redundancy, modified conclusion to reflect the aims the study, and used native English proofreader.

Please use either medical “devices” or “apparatuses”. Don’t mix up.

Response: we updated the manuscript to use the term “medical devices” throughout the manuscript.

The first appearance of the microbe’s name and its abbreviations should be consistent throughout the manuscript. The same should be applied to other abbreviations.

Please check the entire manuscript.

Response: We thank the reviewer for this comment. We ensured consistent usage of the microbe's name and abbreviations throughout the revised manuscript, as well as maintaining uniformity with other abbreviations.

Please follow the scientific way of writing. Materials and methods, results, and discussion should be presented scientifically. Please check the scientific nomenclature of microbes.

Response: We thank the reviewer for the suggestion. We updated the manuscript to restructure the manuscript as per the recommendation.

Methods: For indwelling medical device coating, a cytotoxicity test must be performed to justify its biocompatibility, and it is also good to assess hemocompatibility.

Response: We sincerely appreciate the reviewer's insightful comment. The current study investigates the impact of wettability via plasma surface modification, eliminating the need for any additional coatings. Plasma treatment of medical devices is a widely recognized technique within the medical field, and as such, the authors believe that conducting a cytotoxicity test to confirm biocompatibility is not applicable to this study.

The result is the absolute findings of your experiments. No discussion and no reference should be added.

Response: We thank the reviewer for the comment. The manuscript has been updated following the reviewer’s comment.

In the discussion section, please discuss your result with references in a consistent manner.

Please avoid a discussion that is out of context (not related to your experiment results).

Thanks

Response: L322-328 removed.

Reviewer #2: The authors study the adhesion and biofilm formation of C. albicans on a polymer, which is surface modified to acquire differences in surface wettability. The use of COC as a material to prevent C. albicans biofilm formation seem to be novel and the experiment design and the data to link surface wettability and Candida biofilm growth is interesting. However, it is not clear why several indwelling medical devices are tested, unless the authors had compared them with COC, which was not the case. It is also not clear what materials the medical devices are made of.

Altogether, the authors can remove the information on the medical devices and focus a more rigorous study on the link between surface wettability and C. albicans biofilm formation using COC.

We express our gratitude to the reviewer for their valuable comment, and we extend our apologies for any lack of clarity regarding the rationale for incorporating wettability measurements into the study of commercial indwelling devices. Our objective in conducting surface contact angle tests on these medical devices was to gain insights into the wettability of their internal surfaces.

The outcomes of our investigation revealed that all tested devices exhibited hydrophilic surfaces, which can be surely assumed to create a conducive environment for the attachment of *C. albicans*, consistent with numerous clinical reports of increased resistance due to indwelling devices and as demonstrated in the findings presented within this manuscript with respect to COC. We have taken utmost care to elucidate this point thoroughly in the discussion section of the revised manuscript.

The writing needs to be significantly improved, avoiding repetition of statements, a clear distinction between introduction, methods, results and discussion, and a logical flow of information making it easier to follow.

Response: Thank you for your feedback. We enhanced the writing of the revised manuscript by minimizing repetition, ensuring clear section distinctions, and improving the logical flow for better readability.

The methods section should be more detailed and rigorous. Additionally, the study needs a clear hypothesis and also lack direction.

Response: We appreciate your valuable feedback. We enhanced the methods section in the revised manuscript with more information. Furthermore, we established a clear hypothesis and improved the overall direction of the research for greater clarity and focus.

Please see below additional information

The authors seem to overplay the abstract. Keep it clear and simple and avoid words such as ‘remarkable’, ‘alarming’, ‘breakthrough finding’, and present the facts.

Response: In response to the reviewer's feedback, we have revised the abstract in the updated manuscript to exclude terms such as 'remarkable', 'alarming', and 'breakthrough finding'. Instead, we present only factual information.

Please acknowledge in the introduction other instances where polymers are tested for wettability and Candida adhesion (ex: doi: 10.1128/AEM.71.12.8795-8801.2005)The introduction can focus more on literature information about the impact of surface wettability on C. albicans biofilm development.

Response: We appreciate your valuable suggestion. In the introduction, we acknowledged other instances where polymers were tested for wettability and Candida adhesion, including the reference you provided (doi: 10.1128/AEM.71.12.8795-8801.2005). Furthermore, we focused more on literature information regarding the impact of surface wettability on *C. albicans* biofilm development, thereby enhancing the contextual background of our study.

Please find and summarize more related literature or state the lack of information and therefore, the need for studying it.

Response: The revised manuscript now includes the rationale for conducting this study, stemming from the limited existing literature on the subject. Moreover, it emphasizes the imperative to explore the influence of wettability on *C. albicans* biofilm formation.

Line 71: Please write the full name of the bacteria on their first mention

Response: Corrected in the revised manuscript.

Line 76: Candida need to be italicized throughout the manuscript and attention to detail.

Response: Thank you for pointing this out. This was corrected throughout the revised manuscript.

Line 107: Need more information about fungal cells and their link with surface wettability/contact angles.

Response: Attachment in Candida is governed by several adhesins, including Als1, Als3, Hwp1, and Eap1. These adhesins have been shown to play critical roles in adhesion and biofilm formation. In addition to adhesins, *C. albicans* also produce ECM components, such as β-glucan, chitin, and mannan, which are important for biofilm formation and stability and help to protect the cells within it from host defenses and antifungal agents. In our case, hydrophobic surfaces have been observed to induce a conformational change in fibronectin, resulting in a decrease in cell adhesion. Fibronectin is one of the major mechanisms for adhesion to the fungal cell wall. This is now modified in the introduction and parts of the discussion.

Line 120-124: Clearly state the surfaces the biofilms were developed. The methods need to be rewritten more clearly.

Response: Biofilm growth is exclusively visible within the hydrophilic region as shown in Figure 6 and we added a new section under methods for biofilm formation.

Line 135: What is the substrate used?

Response: Cyclic olefin copolymer (COC).

Figure 1, The schematic diagram and the caption can benefit from explaining upfront the aim of creating two different surfaces on the same substrate and clearly labelling which surface is hydrophobic and which is hydrophilic.

Response: The concept behind incorporating two distinct regions of varying wettability on a single surface is to explore the hypothesis regarding the impact of wettability on *C. albicans* biofilm formation. This notion is visually represented within the manuscript through the inclusion of Figure 5, demonstrating the presence of these distinct regions on the same surface and modifying Figure 5 legend accordingly.

Line 154: O2 – 2 needs to be subscript

Response: This was corrected in the revised manuscript.

195-201: This paragraph can be removed from the results section and similarly avoid repetitive statements. Please present only the results here.

Response: The paragraph was removed from the revised manuscript following the reviewer’s recommendation.

Line 194: This section does not add anything new to the manuscript. Please consider deleting this section. What is the substrate used in this experiment? Candida hyphae formation in the presence of serum and at 37C is well known.

Response: While we concur with the reviewer that the added information did not introduce novel insights, we included this section to validate the successful biofilm formation in our YEPD.

214-223: All this information belongs in the introduction.

Response: The mentioned lines were moved to the introduction part of the revised manuscript.

Line 224-226: Need to describe the methods more clearly.

Response We modified two distinct wettabilities using microfabrication and plasma treatment to selected surface areas and for more details ref doi: 10.3390/polym13142305.

Line 228-231: After washing with DI water, only the hydrophilic area will hold a water droplet? Not clear how that works. Although photographs are shown (need to get more clear close-up photographs), this need to be further characterized and show there is no water in the hydrophilic area.

How much can you tilt the substrate to prevent the water droplet from flowing/falling off the substrate? Need to show better ways of comparatively characterizing the wettability on the two surfaces.

Response: Wettability significantly influences the attachment behavior of water droplets to surfaces. Hydrophilic surfaces encourage water to spread with a small contact angle (<90 degrees), while hydrophobic surfaces cause droplets to bead up with a larger angle (>90 degrees). This attachment process relies on cohesive and adhesive forces, bearing relevance for applications like self-cleaning coatings and biofilm formation. Notably, the authors previously published a study on modifying COC surface wettability using plasma and graphene oxide coating (doi: 10.3390/polym13142305), offering further insights into the COC substrate's wettability.

Surface energy, a key factor, governs the strength of attraction between the surface and water droplets, impacting wetting and spreading. Higher surface energy promotes greater wetting, while lower surface energy results in beading and reduced spreading. The accompanying figure illustrates a COC surface with varied wettabilities, transitioning from high surface energy (where droplets spread) to low surface energy (where droplets do not spread).

In hydrophobic scenarios, water easily falls off the surface. For hydrophilic cases, water falling depends on the interplay of forces between the droplet and the surface.

Line 232: How was Candida grown on the substrate? Please explain this clearly in the methods section.

Response: The candida culture was diluted at a ratio of 1:10 into 50 ml of pre-warmed YEPD medium supplemented with 10% fetal bovine serum (FBS) at 37°C. The resulting cultures were agitated at 200 rpm for a duration of 24 hours, following previously established protocols.

How did you sterilize the substrate prior to Candida growth?

Response: The substrates underwent sterilization by being washed with IPA (Isopropyl Alcohol) and modified in line

Line 256: Line 261-264: How did you perform the XTT assay on the same substrate (hydrophobic and hydrophilic areas)? This is not clearly explained in the methods section. Did you use two separate substrates for the XTT assay?

Response: We modified the methods section to explain that we used two separate substrates for the XTT assay.

Figure 5C is not clear whether it has Candida cells. A light microscopy image will show the cells and hyphae clearly on each surface.

Response: We agree, kindly see the magnified light microscopy, below and can also be seen in Figure 6.

If you are proposing that the COC is a better polymer than the materials used for the commercial devices, then the level of Candida biofilm formation on all the surfaces from each device should be compared with COC. This needs to be quantified using XTT assays and qualitatively observed using microscopy. Additionally, each surface compared to COC should have been tested for surface wettability and Candida biofilm formation and explain the link between surface wettability and Candida biofilm formation. Currently, there is no apparent link between the COC and the medical implants that is tested in the manuscript.

Response: We agree. In this study, we reported COC wettability modifications as a promising approach to modify biofilm formation. In this study, we presented an assessment of the wettability of commercially available indwelling devices. However, it's important to note that our focus did not encompass the investigation of biofilm formation on these devices. The selection of COC as our substrate stemmed from its suitability, owing to the authors' established approach for patterning its wettability and its transparent nature, which facilitates the visualization of yeast on its surface. We acknowledge the points raised by the reviewer as limitations to be addressed in the follow-up study.

Reviewer #3: The paper “Revolutionizing Medical Device Infection Control with Surface Treatment Using Microfabrication” describes using Cyclic olefin copolymer to coat plastic surfaces to evaluate Candida albicans biofilm growth. The authors microfabricated a wafer with patterned wettability, which can influence contact angle. The paper's central idea is how hydrophobicity could be used to avoid or reduce fungi biofilm. While the concept of controlling the wettability of surfaces to prevent biofilm growth has been demonstrated before, the anti-biofilm properties of available commercial medical devices are still an interesting topic. Based on the simplistic model utilized and essential biological questions raised and not answered through the manuscript, I have several concerns that are listed below:

1-The title might be modified once an infection model was not used/developed. Fungi biofilm control might be more appropriate for the title.

Response: We appreciate your valuable suggestion and thank you for your time and effort, we modified the title accordingly.

2- The introduction might be more focused on the polymer and the microfabrication techniques to prevent biofilm formation.

Response: modified, lines 118 to 121

3- Statistics analysis should be used for this study. This is a critical part of a study that involves biofilm formation and growth. What are the tests utilized? Are the tests performed in triplicates? What are the p values of the data found?

Response: We added the following under Statistical analyses. Each experiment was run three times with triplicates each time. A P value of less than 0.05 was regarded as statistically significant for all statistical tests (unpaired t test and correlation coefficient).

4- The controls for this study can be better designed. If the comparison was the current medical devices, why did they not test them for biofilm growth?

Response: We express our gratitude to the reviewer for their valuable comment, and we extend our apologies for any lack of clarity regarding the rationale for incorporating wettability measurements into the study of commercial indwelling devices. Our objective in conducting surface contact angle tests on these medical devices was to gain insights into the wettability of their internal surfaces.

The outcomes of our investigation revealed that all tested devices exhibited hydrophilic surfaces, which can be surely assumed to create a conducive environment for the attachment of C. albicans, consistent with numerous clinical reports of increased resistance due to indwelling devices and as demonstrated in the findings presented within this manuscript with respect to COC. We have taken utmost care to elucidate this point thoroughly in the discussion section of the revised manuscript.

5- In Figure 4, the authors affirmed that increased filamentation was encased within the ECM when cells were treated with serum at 37 °, compared to ECM-coated yeast cells at 30 o. However, the SEM images do not demonstrate significant differences in the biofilm structure.

Response: We agree with the reviewer, however the dimorphic switching under serum and at 37◦C is well documented in the literature. Our goal here is to confirm the successful biofilm formation in YEPD. Furthermore, we changed Figure 4 to reflect a more significant difference in the biofilm structure available to the visual field. This was also confirmed by XTT assay.

6- Figure 5 describes the effect of surface wettability on promoting candida growth. However, any quantitative data was provided to affirm that.

Response: We agree and XTT assay is severe as a semi-quantitative method.

7- In Figure 6, what are the significant differences between the groups?

Response: *P* <0.05
