# Supplementary material for: Transforming medical device biofilm control with surface treatment using microfabrication techniques
Source: PLoS One. 2023 Nov 30;18(11):e0292647. doi: 10.1371/journal.pone.0292647 (PMC10688649; doi:10.1371/journal.pone.0292647)
Supplement: Attachment — Submitted filename: Response to Reviewers 2.docx [file pone.0292647.s002.docx]

Response to Reviewers

Reviewer #1: It is better now and can be accepted for publication. No further comments.

I suggest having a pre-publication proofreading.

Response: We thank Reviewer #1 for time and constructive feedback, and performed the suggested proofreading.

Reviewer #2: Reviewers' Comments are based on the numbering of the version with the tracked changes.

Line 1: Topic should be changed to read better. Please consider removing ‘Revolutionizing”.

Response: modified as requested.

Line 37: found to be?

Response: Yes, and modified as requested.

Line 39: Where was the Candida biofilm growth established? If it is on the medical devices, please mention it. I don’t see any mention about the COC. The data on COC takes up a significant portion of the manuscript and therefore, must be presented in the abstract. The abstract can be better written.

Response: We agree, and modified as requested.

Line 42: C. albicans repetition

Response: Corrected.

Line 73: Still the full scientific name of the microorganisms are not mentioned in the first instance. Here and throughout the manuscript, please pay attention to detail.

Response: Corrected.

In the introduction, need to introduce COC and the rationale for using it to test the wettability. This reviewer thinks the introduction can be trimmed and need more work to give it more focus.

Response: The introduction part of the revised manuscript has been updated as suggested.

Methods: Line 139: The sentence should start by saying “For imaging purposes”? Otherwise, why is it treated with formaldehyde? The methods need to be very clear and logically flow throughout the manuscript.

Response: Modified as suggested.

Line 143-152: The writing is very confusing. This reviewer believes the authors can do a better job in clearly explaining the methodology, here and throughout the methods section. 2,3-bis(2-methoxy-4-nitro-5-sulfophenyl) is mentioned at the beginning and then XTT reagent at the end of the paragraph. Perhaps stick to one chemical nomenclature? Spelling mistakes: XXT, 24 hr. or 24 h. or 24 h please be consistent throughout the manuscript. Need attention to detail.

Response: Modified as suggested.

Statistical analysis section can be the final sub topic in the methods section.

Response: Modified as suggested.

Figure 1E: Can the authors show the hydrophilic and hydrophobic regions in the disc (in the acetone bath) in this image as well, similar to the other ones.

Response: Figure 1 shows the fabrication technique used to pattern the wettability of the COC surface. The hydrophobic and hydrophilic properties are not shown here. Figure 1E shows the wafer in acetone to perform liftoff, the hydrophilic/hydrophobic properties can’t be shown in the figure.

Line 188-191: Do the authors think these sentences belong in the results section together with Figure 2? Perhaps a comparison with the water contact angle measurements of the medical devices? This would be more appropriate.

Response: The wettability of the COC substrate was previously addressed in our earlier publication and is not the central focus of the current manuscript. The authors firmly believe that the placement of these lines within the method section of the manuscript is appropriate, and they should not be considered as part of the results section.

Line 210-211: What are materials that the medical devices are made of? This need to be mentioned.

Response: The materials used and the sterilization method for the four medical devices are

- Butterfly Cannula tubing is made from PVC and sterilized using irradiation.
- Thoracic Catheter is made from clear thermosensitive PVC and sterilized using Ethylene oxide gas.
- Ryle’s Tube is made from medical grade PVC and sterilized by Ethylene oxide gas.
- Foley Catheter is made from latex and sterilized by Ethylene oxide gas.

The above information has been added to Figure 3 of the revised manuscript.

Line 223: Either remove this section or It is better to evaluate the C. albicans biofilm formation on each medical devices (and perhaps if you can compare them with COC - hydrophobic and hydrophilic surfaces)? Perhaps do a XTT assay? This will be a good dataset to compare with the above water contact angle measurements. This will also be a good rationale to further study Wettability and C. albicans biofilm formation on COC in the sections below. And Figure 4 can be moved to a supplementary file.

Response: We agree, and have acknowledged this as a limitation of the current study and have indicated this in the discussion L354.

Line 298 : XXT?

Response: yes and corrected.

Line 244 and Line 275: Change the topic of these two sections. Currently both are very similar.

Response: we agree, and changed the topics accordingly.

Figure 5 and 6: Can you show better quality images, especially Figure 5B and 5C? Also some labelling/indicators (hydrophobic and hydrophilic regions, water droplets, C. albicans growth) would help the readers. Although the information is indicated in the figure caption, labeling some of these information will bring more clarity.

Response: Figure 5 is updated to include labelling and information about the three subfigures. The caption is also updated to include information about the figure.

Line 342-343: The study did not prove the involvement of proteins in the attachment. This statement must be removed.

Response: We agree, and removed.

Line 346-349: These sentences can be trimmed.

Response: Modified as suggested.

Line 362: XXT ? Spelling mistakes and typos here and throughout the manuscript. Need more attention to detail.

Response: corrected.

The story can still be made to flow better with logical flow of information and clear writing. This reviewer believes that the manuscript can still be improved.

Response: The revised manuscript has diligently incorporated all of the suggested changes made by the reviewer. These modifications have greatly enhanced the overall flow of ideas and provided a significant boost in clarity. We extend our heartfelt gratitude to the reviewer for their invaluable and constructive feedback.
